# Nanofluid bioconvection in water-based suspensions containing nanoparticles and oxytactic microorganisms: oscillatory instability

**DOI:** 10.1186/1556-276X-6-100

**Published:** 2011-01-25

**Authors:** Andrey V Kuznetsov

**Affiliations:** 1 Dept. of Mechanical and Aerospace Engineering, North Carolina State University, Campus Box 7910, Raleigh, NC 27695-7910, USA

## Abstract

The aim of this article is to propose a novel type of a nanofluid that contains both nanoparticles and motile (oxytactic) microorganisms. The benefits of adding motile microorganisms to the suspension include enhanced mass transfer, microscale mixing, and anticipated improved stability of the nanofluid. In order to understand the behavior of such a suspension at the fundamental level, this article investigates its stability when it occupies a shallow horizontal layer. The oscillatory mode of nanofluid bioconvection may be induced by the interaction of three competing agencies: oxytactic microorganisms, heating or cooling from the bottom, and top or bottom-heavy nanoparticle distribution. The model includes equations expressing conservation of total mass, momentum, thermal energy, nanoparticles, microorganisms, and oxygen. Physical mechanisms responsible for the slip velocity between the nanoparticles and the base fluid, such as Brownian motion and thermophoresis, are accounted for in the model. An approximate analytical solution of the eigenvalue problem is obtained using the Galerkin method. The obtained solution provides important physical insights into the behavior of this system; it also explains when the oscillatory mode of instability is possible in such system.

## Introduction

The term "nanofluid" was coined by Choi in his seminal paper presented in 1995 at the ASME Winter Annual Meeting [1]. It refers to a liquid containing a dispersion of submicronic solid particles (nanoparticles) with typical length on the order of 1-50 nm [2]. The unique properties of nanofluids include the impressive enhancement of thermal conductivity as well as overall heat transfer [3-7]. Various mechanisms leading to heat transfer enhancement in nanofluids are discussed in numerous publications; see, for example [8-12].

Wang [13-15] pioneered in developing the constructal approach, created by Bejan [16-19], for designing nanofluids. Nanofluids enhance the thermal performance of the base fluid; the utilization of the constructal theory makes it possible to design a nanofluid with the best microstructure and performance within a specified type of microstructures.

Recent publications show significant interest in applications of nanofluids in various types of microsystems. These include microchannels [20], microheat pipes [21], microchannel heat sinks [22], and microreactors [23]. There is also significant potential in using nanomaterials in different bio-microsystems, such as enzyme biosensors [24]. In [25], the performance of a bioseparation system for capturing nanoparticles was simulated. There is also strong interest in developing chip-size microdevices for evaluating nanoparticle toxicity; Huh et al. [26] suggested a biomimetic microsystem that reconstitutes the critical functional alveolar-capillary interface of the human lung to evaluate toxic and inflammatory responses of the lung to silica nanoparticles.

The aim of this article is to propose a novel type of a nanofluid that contains both nanoparticles and oxytactic microorganisms, such as a soil bacterium *Bacillus subtilis*. These particular microorganisms are oxygen consumers that swim up the oxygen concentration gradient. There are important similarities and differences between nanoparticles and motile microorganisms. In their impressive review of nanofluids research, Wang and Fan [27] pointed out that nanofluids involve four scales: the molecular scale, the microscale, the macroscale, and the megascale. There is interaction between these scales. For example, by manipulating the structure and distribution of nanoparticles the researcher can impact macroscopic properties of the nanofluid, such as its thermal conductivity. Similar to nanofluids, in suspensions of motile microorganisms that exhibit spontaneous formation of flow patterns (this phenomenon is called bioconvection) physical laws that govern smaller scales lead to a phenomenon visible on a larger scale. While superfluidity and superconductivity are quantum phenomena visible at the macroscale, bioconvection is a mesoscale phenomenon, in which the motion of motile microorganisms induces a macroscopic motion (convection) in the fluid. This happens because motile microorganisms are heavier than water and they generally swim in the upward direction, causing an unstable top-heavy density stratification which under certain conditions leads to the development of hydrodynamic instability. Unlike motile microorganisms, nanoparticles are not self-propelled; they just move due to such phenomena as Brownian motion and thermophoresis and are carried by the flow of the base fluid. On the contrary, motile microorganisms can actively swim in the fluid in response to such stimuli as gravity, light, or chemical attraction. Combining nanoparticles and motile microorganisms in a suspension makes it possible to use benefits of both of these microsystems.

One possible application of bioconvection in bio-microsystems is for mass transport enhancement and mixing, which are important issues in many microsystems [28,29]. Also, the results presented in [30] suggest using bioconvection in a toxic compound sensor due to the ability of some toxic compounds to inhibit the flagella movement and thus suppress bioconvection. Also, preventing nanoparticles from agglomerating and aggregating remains a significant challenge. One of the reasons why this is challenging is because although inducing mixing at the macroscale is easy and can be achieved by stirring, inducing and controlling mixing at the microscale is difficult. Bioconvection can provide both types of mixing. Macroscale mixing is provided by inducing the unstable density stratification due to microorganisms' upswimming. Mixing at the microscale is provided by flagella (or flagella bundle) motion of individual microorganisms. Due to flagella rotation, microorganisms push fluid along their axis of symmetry, and suck it from the sides [31]. While the estimates given in [32] show that the stresslet stress produced by individual microorganisms have negligible effect on macroscopic motion of the fluid (which is rather driven by the buoyancy force induced by the top-heavy density stratification due to microorganisms' upswimming), the effect produced by flagella rotation is not negligible on the microscopic scale (on the scale of a microorganism and a nanoparticle).

In order to use suspensions containing both nanoparticles and motile microorganisms in microsystems, the behavior of such suspensions must be understood at the fundamental level. Bio-thermal convection caused by the combined effect of upswimming of oxytacic microorganisms and temperature variation was investigated in [33-36]. Bioconvection in nanofluids is expected to occur if the concentration of nanoparticles is small, so that nanoparticles do not cause any significant increase of the viscosity of the base fluid. The problem of bioconvection in suspensions containing small solid particles (nanoparticles) was first studied in [37-41] and then recently in [42]. Non-oscillatory bioconvection in suspensions of oxytactic microorganisms was considered in Kuznetsov AV: **Nanofluid bioconvection: Interaction of microorganisms oxytactic upswimming, nanoparticle distribution and heating/cooling from below**. *Theor Comput Fluid Dyn *2010, submitted. This article extends the theory to the case of oscillatory convection in suspensions containing both nanoparticles and oxytactic microorganisms.

### Governing equations

The governing equations are formulated for a water-based nanofluid containing nanoparticles and oxytactic microorganisms. The nanofluid occupies a horizontal layer of depth *H*. It is assumed that the nanoparticle suspension is stable. According to Choi [2], there are methods (including suspending nanoparticles using either surfactant or surface charge technology) that lead to stable nanofluids. It is further assumed that the presence of nanoparticles has no effect on the direction of microorganisms' swimming and on their swimming velocity. This is a reasonable assumption if the nanoparticle suspension is dilute; the concentration of nanoparticles has to be small anyway for the bioconvection-induced flow to occur (otherwise, a large concentration of nanoparticles would result in a large suspension viscosity which would suppress bioconvection).

In formulating the governing equations, the terms pertaining to nanoparticles are written using the theory developed in Buongiorno [43], while the terms pertaining to oxytactic microorganisms are written using the approach developed by Hillesdon and Pedley [44,45].

The continuity equation for the nanoparticle-microorganism suspension considered in this research is

(1) ∇⋅U=0

where U = (*u,v,w*) is the dimensionless nanofluid velocity, defined as U*H/*α*_f_; U* is the dimensional nanofluid velocity; *α*_f _is the thermal diffusivity of a nanofluid, *k*/(*ρc*)_f_; *k *is the thermal conductivity of the nanofluid; and (*ρc*)_f _is the volumetric heat capacity of the nanofluid. The dimensionless coordinates are defined as (*x,y,z*) = (x*, y*, z*)/*H*, where *z *is the vertically downward coordinate.

The buoyancy force can be considered to be made up of three separate components that result from: the temperature variation of the fluid, the nanoparticle distribution (nanoparticles are heavier than water), and the microorganism distribution (microorganisms are also heavier than water). Utilizing the Boussinesq approximation (which is valid because the inertial effects of the density stratification are negligible, the dominant term multiplying the inertia terms is the density of the base fluid that exceeds by far the density stratification), the momentum equation can be written as:

(2) $$\frac{1}{Pr}\left(\frac{\partial U}{\partial t}+U\cdot \nabla U\right)=-\nabla p+\nabla^{2}U+Rm\;\hat{k}-RaT\hat{k}+Rn\phi \hat{k}+\frac{Rb}{Lb}n\hat{k}$$

where $\hat{k}$ is the vertically downward unit vector.

The dimensionless variables in Equation 2 are defined as:

(3) $$t=t^{*}\alpha_{\text{f}}/H^{2},\;p=p^{*}H^{2}/\mu \alpha_{\text{f}},\;\phi =\frac{\phi^{*}-\phi_{0}^{*}}{\phi_{1}^{*}-\phi_{0}^{*}},\;T=\frac{T^{*}-T_{c}^{*}}{T_{h}^{*}-T_{c}^{*}},\;n=n^{*}/n_{0}^{*}$$

where *t *is the dimensionless time, *p *is the dimensionless pressure, *ϕ *is the relative nanoparticle volume fraction, *T *is the dimensionless temperature, *n *is the dimensionless concentration of microorganisms, t* is the time, p^* ^is the pressure, *μ *is the viscosity of the suspension (containing the base fluid, nanoparticles and microorganisms), ϕ^* ^is the nanoparticle volume fraction, $\phi_{0}^{*}$ is the nanoparticle volume fraction at the lower wall, $\phi_{1}^{*}$ is the nanoparticle volume fraction at the upper wall, T* is the nanofluid temperature, $T_{c}^{*}$ is the temperature at the upper wall (also used as a reference temperature), $T_{h}^{*}$ is the temperature at the lower wall, n* is the concentration of microorganisms, and $n_{0}^{*}$ is the average concentration of microorganisms (concentration of microorganisms in a well-stirred suspension).

The dimensionless parameters in Equation 2, namely, the Prandtl number, *Pr*; the basic-density Rayleigh number, *Rm*; the traditional thermal Rayleigh number, *Ra*; the nanoparticle concentration Rayleigh number, *Rn*; the bioconvection Rayleigh number, *Rb*; and the bioconvection Lewis number, *Lb*, are defined as follows:

(4) $$Pr=\frac{\mu }{\rho_{\text{f0}}\alpha_{\text{f}}},\;Rm=\frac{\left[\rho_{\text{p}}\phi_{0}^{*}+\rho_{\text{f0}}(1-\phi_{0}^{*})\right]gH^{3}}{\mu \alpha_{\text{f}}},\;Ra=\frac{\rho_{\text{f0}}g\beta H^{3}\left(T_{h}^{*}-T_{c}^{*}\right)}{\mu \alpha_{\text{f}}}$$

(5) $$Rn=\frac{(\rho_{\text{p}}-\rho_{\text{f0}})(\phi_{1}^{*}-\phi_{0}^{*})gH^{3}}{\mu \alpha_{\text{f}}},\;Rb=\frac{\Delta \rho g\theta n_{0}^{*}H^{3}}{\mu D_{\text{mo}}},\;Lb=\frac{\alpha_{\text{f}}}{D_{\text{mo}}}$$

where *ρ*_*f0 *_is the base-fluid density at the reference temperature; *ρ*_*p *_is the nanoparticle mass density; *g *is the gravity; *β *is the volumetric thermal expansion coefficient of the base fluid; Δ*ρ *is the density difference between microorganisms and a base fluid, *ρ*_mo _*- ρ*_f0_; *ρ*_mo _is the microorganism mass density; *θ *is the average volume of a microorganism; and *D*_mo _is the diffusivity of microorganisms (in this model, following [44,45], all random motions of microorganisms are simulated by a diffusion process).

The conservation equation for nanoparticles contains two diffusion terms on the right-hand side, which represent the Brownian diffusion of nanoparticles and their transport by thermophoresis (a detailed derivation is available in [43,46]):

(6) $$\frac{\partial \phi }{\partial t}+U\cdot \nabla \phi =\frac{1}{Ln}\nabla^{2}\phi +\frac{N_{A}}{Ln}\nabla^{2}T$$

In Equation 6, the nanoparticle Lewis number, *Ln*, and a modified diffusivity ratio, *N*_*A *_(this parameter is somewhat similar to the Soret parameter that arises in cross-diffusion phenomena in solutions), are defined as:

(7) $$Ln=\frac{\alpha_{\text{f}}}{D_{\text{B}}},\;N_{A}=\frac{D_{\text{T}}\left(T_{h}^{*}-T_{c}^{*}\right)}{D_{\text{B}}T_{c}^{*}(\phi_{1}^{*}-\phi_{0}^{*})}$$

where *D*_B _is the Brownian diffusion coefficient of nanoparticles and *D*_T _is the thermophoretic diffusion coefficient.

The right-hand side of the thermal energy equation for a nanofluid accounts for thermal energy transport by conduction in a nanofluid as well as for the energy transport because of the mass flux of nanoparticles (again, a detailed derivation is available in [43,46]):

(8) $$\frac{\partial T}{\partial t}+U\cdot \nabla T=\nabla^{2}T+\frac{N_{B}}{Ln}\nabla \phi \cdot \nabla T+\frac{N_{A}N_{B}}{Ln}\nabla T\cdot \nabla T$$

In Equation 8, *N*_*B *_is a modified particle-density increment, defined as:

(9) $$N_{B}=\frac{{\left(\rho c\right)}_{\text{p}}}{{\left(\rho c\right)}_{\text{f}}}\left(\phi_{1}^{*}-\phi_{0}^{*}\right)$$

where (*ρc*)_p _is the volumetric heat capacity of the nanoparticles.

The right-hand side of the equation expressing the conservation of microorganisms describes three modes of microorganisms transport: due to macroscopic motion (convection) of the fluid, due to self-propelled directional swimming of microorganisms relative to the fluid, and due diffusion, which approximates all stochastic motions of microorganisms:

(10) $$\frac{\partial n}{\partial t}=-\nabla \cdot \left(nU+nV-\frac{1}{Lb}\nabla n\right)$$

where **V **is the dimensionless swimming velocity of a microorganism, V*H/*α*_f_, which is calculated as [44,45]:

(11) $$V=\frac{Pe}{Lb}\hat{H}\left(C\right)\nabla C$$

In Equation 11 $\hat{H}$ is the Heaviside step function and *C *is the dimensionless oxygen concentration, defined as:

(12) $$C=\frac{C^{*}-C_{\min}^{*}}{C_{0}^{*}-C_{\min}^{*}}$$

where C* is the dimensional oxygen concentration, $C_{0}^{*}$ is the upper-surface oxygen concentration (the upper surface is assumed to be open to atmosphere), and $C_{\min}^{*}$ is the minimum oxygen concentration that microorganisms need to be active. Equation 11 thus assumes that microorganisms swim up the oxygen concentration gradient and that their swimming velocity is proportional to that gradient; however, in order for microorganisms to be active the oxygen concentration need to be above $C_{\min}^{*}$. Since this article deals with a shallow layer situation, it is assumed that $C^{*}>C_{\min}^{*}$ throughout the layer thickness, and the Heaviside step function, $\hat{H}\left(C\right)$, in Equation 11 is equal to unity.

Also, the bioconvection Péclet number, *Pe*, in Equation 11 is defined as:

(13) $$Pe=\frac{bW_{\text{mo}}}{D_{\text{mo}}}$$

where *b *is the chemotaxis constant (which has the dimension of length) and *W*_mo _is the maximum swimming speed of a microorganism (the product *bW*_mo _is assumed to be constant).

Finally, the oxygen conservation equation is:

(14) $$\frac{\partial C}{\partial t}+U\cdot \nabla C=\frac{\text{1}}{Le}\nabla^{2}C-\hat{\beta }n$$

The first term on the right-hand side of Equation 14 represents oxygen diffusion, while the second term represents oxygen consumption by microorganisms.

The new dimensionless parameters in Equation 14 are

(15) $$Le=\frac{\alpha_{\text{f}}}{D_{S}},\;\hat{\beta }=\frac{\gamma H^{2}n_{0}^{*}}{\left(C_{0}^{*}-C_{\min}^{*}\right)\;\alpha_{\text{f}}}$$

where *Le *is the traditional Lewis number, $\hat{\beta }$ is the dimensionless parameter describing oxygen consumption by the microorganisms, *D*_*S *_is the diffusivity of oxygen, and *γ *is a dimensional constant describing consumption of oxygen by the microorganisms.

According to Hillesdon and Pedley [45], the layer can be treated as shallow as long as the following condition is satisfied:

(16) $$H\leq {\left(\frac{2{\left(\exp\left(Pe\right)-1\right)}^{1/2}}{Pe\;Le}\frac{\left(C_{0}^{*}-C_{\min}^{*}\right)\;\alpha_{f}}{\gamma n_{0}^{*}}\tan^{-1}\left[{\left(\exp\left(Pe\right)-1\right)}^{1/2}\right]\right)}^{1/2}$$

Equation 16 gives the maximum layer depth for which the oxygen concentration at the bottom does not drop below $C_{\min}^{*}$.

The boundary conditions for Equations 1, 2, 6, 8, 10, and 14 are imposed as follows. It is assumed that the temperature and the volumetric fraction of the nanoparticles are constant on the boundaries and the flux of microorganisms through the boundaries is equal to zero. The lower boundary is always assumed rigid and the upper boundary can be either rigid or stress-free. The boundary conditions for case of a rigid upper wall are

(17) $$w=0,\;\frac{\partial w}{\partial z}=\text{0,}\;T=1,\;\phi =0,\;\frac{\text{d}n}{\text{d}z}=\text{0,}\;\frac{\partial C}{\partial z}=\text{0}\;\text{at }z=1\;\left(\text{the lower wall}\right)$$

(18) $$w=0,\;\frac{\partial w}{\partial z}=\text{ 0,}\;T=0,\;\phi =1,\;Pe\;n\frac{\text{d}C}{\text{d}z}-\frac{\text{d}n}{\text{d}z}=\text{0,}\;C=\text{1}\;\text{at }z=0\;\;\left(\text{the upper wall}\right)$$

The fifth equation in (18) is equivalent to the statement that the total flux of microorganisms at the upper surface is equal to zero: the microorganisms swim vertically upward at the top surface but (because their concentration gradient at the top surface is directed vertically upward) they are simultaneously pushed downward by diffusion; the two fluxes are equal but opposite in direction).

If the upper surface is stress-free, the second equation in (18) is replaced with the following equation:

(19) $$\frac{\partial^{2}w}{\partial z^{2}}=\text{ 0}$$

### Basic state

The solution for the basic state corresponds to a time-independent quiescent situation. The solution is of the following form:

(20) $$U_{\text{b}}=0,\;p=p_{\text{b}}\text{(}z\text{),}\;T=T_{\text{b}}(z),\;\phi =\phi_{\text{b}}\text{(}z\text{),}\;n=n_{\text{b}}\text{(}z\text{),}\;C=C_{\text{b}}\left(z\right)$$

In this case, the solution of Equations 6, 8, 10, and 14 subjects to boundary conditions (17) and (18) is (the particular form of hydrodynamic boundary conditions at the upper surface is not important because the solution in the basic state is quiescent):

(21) $$\phi_{\text{b}}\left(z\right)=-N_{A}\frac{\exp\left[\frac{\left(1-N_{A}\right)N_{B}}{Ln}z\right]-1}{\exp\left[\frac{\left(1-N_{A}\right)N_{B}}{Ln}\right]-1}-(1-N_{A})z+1$$

(22) $$T_{\text{b}}\left(z\right)=\frac{\exp\left[\frac{\left(1-N_{A}\right)N_{B}}{Ln}z\right]-1}{\exp\left[\frac{\left(1-N_{A}\right)N_{B}}{Ln}\right]-1}$$

(23) $$n_{\text{b}}\left(z\right)=\frac{A_{1}^{2}}{\text{2}Pe\;\hat{\beta }Le}\sec^{2}\left(\frac{A_{1}\left(1-z\right)}{2}\right)$$

(24) $$C_{\text{b}}\left(z\right)=1-\frac{2}{Pe}\ln\left(\frac{\cos\left\{A_{1}\left(1-z\right)/2\right\}}{\cos\left\{A_{1}/2\right\}}\right)$$

where *A*_1 _is the smallest positive root of the transcendental equation

(25) $$\tan\left(\frac{A_{1}}{2}\right)=Pe\frac{\hat{\beta }Le}{A_{1}}$$

The solutions given by Equations 23 and 24 were first reported in [44].

The pressure distribution in the basic state, *p*_b _(*z*), can then be obtained by integrating the following form of the momentum equation (which follows from Equation 2):

(26) $$-\frac{\text{d}p_{\text{b}}}{\text{d}z}+Rm-Ra\;T_{\text{b}}+Rn\;\phi_{\text{b}}+\frac{Rb}{Lb}n_{\text{b}}=0$$

Equations 21 and 22 can be simplified if characteristic parameter values for a typical nanofluid are considered. Based on the data presented in Buongiorno [43] for an alumina/water nanofluid, the following dimensional parameter values are utilized: $\phi_{0}^{*}=0.01$, *α*_f _= 2 × 10^-7^m^2^/s, *D*_B _= 4 × 10^-11^m^2^/s, *μ *= 10^-3 ^Pas, and *ρ*_f0 _= 10^3 ^kg/m^3^. The thermophoretic diffusion coefficient, *D*_T_, is estimated as $\tau \frac{\mu }{\rho }\phi_{0}^{*}$, where, according to Buongiorno [43], *τ *is estimated as 0.006. This results in *D*_T _= 6 × 10^-11^m^2^/s. The nanoparticle Lewis number is then estimated as *Ln *= 5.0 × 10^3^. The modified diffusivity ratio, *N*_*A*_, and the modified particle-density increment, *N*_*B*_, depend on the temperature difference between the lower and the upper plates and on the nanoparticle fraction decrement. Assuming that $T_{h}^{*}-T_{c}^{*}=1\;\;\text{K}$, $\phi_{1}^{*}-\phi_{0}^{*}=0.001$, and $T_{c}^{*}=300\;\;\text{K}$, gives the following estimates: *N*_*A *_= 5 and *N*_*B *_= 7.5 × 10^-4^. This suggests that the exponents in Equations 21 and 22 are small and that these equations can be simplified as:

(27) $$\phi_{\text{b}}\left(z\right)=1-z$$

(28) $$T_{\text{b}}\left(z\right)=z$$

### Linear instability analysis

Perturbations are superimposed on the basic solution, as follows:

(29) $$\begin{array}{l} \left[U,T,\phi ,n,C,p\right]=\left[0,T_{\text{b}}\left(z\right),\phi_{\text{b}}\left(z\right),n_{\text{b}}\left(z\right),C_{\text{b}}\left(z\right),p_{\text{b}}\left(z\right)\right]\\ \;+\varepsilon \left[U^{\prime }\left(t,x,y,z\right),T^{\prime }\left(t,x,y,z\right),\phi^{\prime }\left(t,x,y,z\right),n^{\prime }\left(t,x,y,z\right),C^{\prime }\left(t,x,y,z\right),p^{\prime }\left(t,x,y,z\right)\right] \end{array}$$

Equation 29 is then substituted into Equations 1, 2, 6, 8, 10, and 14, the resulting equations are linearized and the use is made of Equations 27 and 28. This procedure results in the following equations for the perturbation quantities:

(30) $$\nabla \cdot U^{\prime }=0$$

(31) $$\frac{1}{Pr}\frac{\partial U^{\prime }}{\partial t}=-\nabla p^{\prime }+\nabla^{2}U^{\prime }-RaT^{\prime }\hat{k}+Rn\phi^{\prime }\hat{k}+\frac{Rb}{Lb}n^{\prime }\hat{k}$$

(32) $$\frac{\partial T^{\prime }}{\partial t}+w^{\prime }=\nabla^{2}T^{\prime }+\frac{N_{B}}{Ln}\left(\frac{\partial \phi^{\prime }}{\partial z}-\frac{\partial T^{\prime }}{\partial z}\right)+\frac{2N_{A}N_{B}}{Ln}\frac{\partial T^{\prime }}{\partial z}$$

(33) $$\frac{\partial \phi^{\prime }}{\partial t}-w^{\prime }=\frac{1}{Ln}\nabla^{2}\phi^{\prime }+\frac{N_{A}}{Ln}\nabla^{2}T^{\prime }$$

(34) $$\frac{\partial n^{\prime }}{\partial t}+w^{\prime }\frac{dn_{\text{b}}}{dz}+\frac{Pe}{Lb}\left(\frac{\partial C^{\prime }}{\partial z}\frac{dn_{\text{b}}}{dz}+\frac{dC_{\text{b}}}{dz}\frac{\partial n^{\prime }}{\partial z}+n^{\prime }\frac{d^{2}C_{\text{b}}}{dz^{2}}+n_{\text{b}}\nabla^{2}C^{\prime }\right)=\frac{1}{Lb}\nabla^{2}n^{\prime }$$

(35) $$\frac{\partial C^{\prime }}{\partial t}+w^{\prime }\frac{\text{d}C_{\text{b}}}{\text{d}z}=\frac{\text{1}}{Le}\nabla^{2}C^{\prime }-\hat{\beta }n^{\prime }$$

Equations 30 to 35 are independent of *Rm *since this parameter is just a measure of the basic static pressure gradient. In order to eliminate the pressure and horizontal components of velocity from Equations 30 and 31, Equation 31 (see [46]) is operated with $\hat{k}\cdot \text{curl curl}$ and the use is made of the identity curl curl ≡ grad div - ∇^2 ^together with Equation 30. This results in the reduction of Equations 30 and 31 to the following scalar equation which involves only one component of the perturbation velocity, *w*':

(36) $$\frac{1}{Pr}\frac{\partial }{\partial t}\nabla^{2}w^{\prime }-\nabla^{4}w^{\prime }=-Ra\nabla_{\text{H}}^{2}T^{\prime }+Rn\nabla_{\text{H}}^{2}\phi^{\prime }+\frac{Rb}{Lb}\nabla_{\text{H}}^{2}n^{\prime }$$

where $\nabla_{\text{H}}^{2}$ is the two-dimensional Laplacian operator in the horizontal plane and ∇^4^*w' *is the Laplacian of the Laplacian of *w'*.

Equations 17 and 18 then lead to the following boundary conditions for the perturbation quantities for the case when both the lower and upper walls are rigid:

(37) $$w^{\prime }=0,\;\frac{\partial w^{\prime }}{\partial z}=\text{0,}\;T^{\prime }=0,\;\phi^{\prime }=0,\;\frac{\text{d}n^{\prime }}{\text{d}z}=\text{0,}\;\frac{\text{d}C^{\prime }}{\text{d}z}=\text{0}\;\text{at}\;z=1\;\left(\text{the lower wall}\right)$$

(38) $$w^{\prime }=0,\;\frac{\partial w^{\prime }}{\partial z}=\text{0,}\;T^{\prime }=0,\;\phi^{\prime }=0,\;Pe\left[n_{\text{b}}\frac{\text{d}C^{\prime }}{\text{d}z}+\frac{\text{d}C_{\text{b}}}{\text{d}z}n^{\prime }\right]-\frac{\text{d}n^{\prime }}{\text{d}z}=\text{0,}\;C^{\prime }=\text{0}\;\text{at}\;z=0\;\left(\text{the upper wall}\right)$$

If the upper boundary is stress-free, the second equation in Equation 38 is replaced by

(39) $$\frac{\partial^{2}w^{\prime }}{\partial z^{2}}=\text{0}\;\text{at }z=0$$

The method of normal modes is used to solve a linear boundary-value problem composed of differential Equations 32 to 36 and boundary conditions (37), (38) (or (39)). A normal mode expansion is introduced as:

(40) $$\left[w^{\prime },T^{\prime },\phi^{\prime },n^{\prime },C^{\prime }\right]=\left[W(z),\Theta (z),\Phi (z),N\left(z\right),\Xi \left(z\right)\right]f\left(x,y\right)\exp(st),$$

where the function *f*(*x,y*) satisfies the following equation:

(41) $$\frac{\partial^{2}f}{\partial x^{2}}+\frac{\partial^{2}f}{\partial y^{2}}=-m^{2}f$$

and *m *is the dimensionless horizontal wavenumber.

Substituting Equation 40 into Equations 36 and 32 to 35, utilizing Equation 41, and letting $\Xi \to \hat{\beta }\;\bar{\Xi }$ (so that the resulting equation for amplitudes would depend on the product $\varpi =Pe\hat{\beta }$ rather than on *Pe *and $\hat{\beta }$ individually), the following equations for the amplitudes, *W*, Θ, Φ, *N*, and $\bar{\Xi }$, are obtained:

(42) $$\frac{{\text{d}}^{4}W}{\text{d}z^{4}}-2m^{2}\frac{{\text{d}}^{2}W}{\text{d}z^{2}}+m^{4}W-\frac{s}{Pr}\frac{{\text{d}}^{2}W}{\text{d}z^{2}}+m^{2}\frac{s}{\mathrm{Pr}}W+Ra\;m^{2}\Theta -Rn\;m^{2}\Phi -\frac{Rb}{Lb}m^{2}N=0$$

(43) $$-W+\frac{{\text{d}}^{2}\Theta }{\text{d}z^{2}}-\frac{N_{B}}{Ln}\frac{\text{d}\Theta }{\text{d}z}+\frac{2N_{A}N_{B}}{Ln}\frac{\text{d}\Theta }{\text{d}z}-\left(m^{2}+s\right)\Theta +\frac{N_{B}}{Ln}\frac{\text{d}\Phi }{\text{d}z}=0$$

(44) $$-W+\frac{N_{A}}{Ln}m^{2}\Theta +\frac{1}{Ln}m^{2}\Phi +s\Phi -\frac{N_{A}}{Ln}\frac{{\text{d}}^{2}\Theta }{\text{d}z^{2}}-\frac{1}{Ln}\frac{{\text{d}}^{2}\Phi }{\text{d}z^{2}}=0$$

(45) $$\begin{array}{l} -2A_{1}\;Le\;\varpi \tan\left[\frac{1}{2}A_{1}\left(1-z\right)\right]\frac{\text{d}N}{\text{d}z}-A_{1}^{3}\;\sec^{2}\left[\frac{1}{2}A_{1}\left(1-z\right)\right]\tan\left[\frac{1}{2}A_{1}\left(1-z\right)\right]\left(Lb\;W+\varpi \frac{\text{d}\bar{\Xi }}{\text{d}z}\right)\\ +2\;Le\;\varpi \left(m^{2}N-\frac{{\text{d}}^{2}N}{\text{d}z^{2}}\right)+A_{1}^{2}\;\varpi \sec^{2}\left[\frac{1}{2}A_{1}\left(1-z\right)\right]\left(Le\;N-m^{2}\bar{\Xi }+\frac{{\text{d}}^{2}\bar{\Xi }}{\text{d}z^{2}}\right)+2Lb\;Le\;\varpi \;s\;N=0 \end{array}$$

(46) $$\varpi N-A_{1}\tan\left(\frac{1}{2}A_{1}\left(1-z\right)\right)W+\frac{\varpi m^{2}\bar{\Xi }}{\text{Le}}+\varpi s\;\bar{\Xi }-\frac{\varpi }{Le}\frac{{\text{d}}^{2}\bar{\Xi }}{\text{d}z^{2}}=0$$

where Equation 25 for *A*_1 _is reduced to

(47) $$\tan\left(\frac{A_{1}}{2}\right)=\frac{\varpi Le}{A_{1}}$$

In Equations 42 to 46 *s *is a dimensionless growth factor; for neutral stability the real part of *s *is zero, so it is written *s *= *iω*, where *ω *is a dimensionless frequency (it is a real number).

For the case of rigid-rigid walls, the boundary conditions for the amplitudes are

(48) $$W=0,\;\frac{\text{d}W}{\text{d}z}=\text{0,}\;\Theta =0,\;\Phi =0,\;\frac{\text{d}N}{\text{d}z}=\text{0,}\;\frac{\text{d}\bar{\Xi }}{\text{d}z}=\text{0}\;\text{at}\;z=1\;\left(\text{the lower wall}\right)$$

(49) $$W=0,\;\frac{\text{d}W}{\text{d}z}=\text{0,}\;\Theta =0,\;\Phi =0,\;\;{\varpi n_{\text{b}}|}_{z=0}\frac{\text{d}\bar{\Xi }}{\text{d}z}+Pe{\frac{\text{d}C_{\text{b}}}{\text{d}z}|}_{z=0}\;N-\frac{\text{d}N}{\text{d}z}=\text{0,}\;\bar{\Xi }=\text{0}\;\text{at}\;z=0\;\left(\text{the upper wall}\right)$$

If the upper surface is stress-free, the second equation in (49) is replaced by

(50) $$\frac{{\text{d}}^{2}W}{\text{d}z^{2}}=\text{0}\;\text{at}\;z=0$$

Equations 42 to 46 are solved by a single-term Galerkin method. For the case of the rigid-rigid boundaries, the trial functions, which satisfy the boundary conditions given by Equations 48 and 49, are

(51) $$W_{1}=z^{2}{\left(1-z\right)}^{2},\;\Theta_{1}=z(1-z),\;\Phi_{1}=z(1-z),\;N_{1}=1+\alpha \left(z-\frac{1}{2}z^{2}\right),\;{\bar{\Xi }}_{1}=z-\frac{1}{2}z^{2}$$

where

(52) $$\alpha =\frac{A_{1}\left(A_{1}-Le\;\sin A_{1}\right)}{Le\left(1+\cos A_{1}\right)}$$

and *A*_1 _is given by Equation 47.

If the upper boundary is stress-free, *W*_1 _is replaced by

(53) $$W_{1}=z-3z^{3}+2z^{4}$$

and the rest of the trial functions are still given by Equation 51. *W*_1 _given by Equation 53 satisfies the boundary condition given by Equation 50.

## Results and discussion

### Rigid-rigid boundaries

For the case of the rigid-rigid boundaries the utilization of a standard Galerkin procedure (see, for example [47]), which involves substituting the trial functions given by Equation 51 into Equations 42 to 46, calculating the residuals, and making the residuals orthogonal to the relevant trial functions, results in the following eigenvalue equation relating three Rayleigh numbers, *Ra*, *Rn*, and *Rb*:

(54) $$F_{1}Ra+F_{2}Rn+F_{3}Rb-F_{4}=0$$

where functions *F*_1_, *F*_2_, *F*_3_, and *F*_4 _are given in the appendix [see Equations A1 to A4], they depend on *Lb*, *Le*, *Ln*, *Pr*, *N*_*A*_, *ϖ*, *ω*, and *m*. It is interesting that Equation 54 is independent of *N*_*B *_at this order (one-term Galerkin) of approximation.

In order to evaluate the accuracy of the one-term Galerkin approximation used in obtaining Equation 54 the accuracy of this equation is estimated for the case of non-oscillatory instability (which corresponds to *ω *= 0) for the situation when the suspension contains no microorganisms (this corresponds to $n_{0}^{*}=0$, which leads to *Rb *= 0) and no nanoparticles (this leads to *Rn *= 0).

In this limiting case Equation 54 collapses to

(55) $$Ra=\frac{28\left(10+m^{2}\right)\left(504+24m^{2}+m^{4}\right)}{27m^{2}}$$

The right-hand side of Equation 55 takes the minimum value of 1750 at *m*_c _= 3.116; the obtained critical value of *Ra *is 2.5% greater than the exact value (1707.762) for this problem reported in [48]. The corresponding critical value of the wavenumber is 0.03% smaller than the exact value (3.117) reported in [48].

Based on the data presented in [44,45] for soil bacterium *Bacillus subtilis*, the following parameter values for these microorganisms are used: *D*_m _= 1.3 × 10^-10 ^m^2^/s, *D*_s _= 2.12 × 10^-9 ^m^2^/s, Δ*ρ *= 100 kg/m^3^, $n_{0}^{*}=10^{15}\;{\text{cells/m}}^{\text{3}}$, *θ *= 10^-18 ^m^3^, and *H *= 2.5 × 10^-3 ^m (or 2.5 mm, this is a typical depth of a shallow layer; this size is also typical for a microdevice). Also, according to Hillesdon et al. [45], for *Bacillus subtilis *dimensionless parameters can be estimated as follows: *Pe *= 15*H*, $\hat{\beta }\;=7H^{2}/\text{Le}$, where the layer depth, *H*, must be given in mm. Based on [43], the following parameter values for a typical alumina/water nanofluid are utilized: $\phi_{0}^{*}=0.01$, *ρ*_f0 _= 10^3 ^kg/m^3^, *ρ*_p _= 4 × 10^3 ^kg/m^3^, (*ρc*)_p _= 3.1 × 10^6 ^J/m^3^, *α*_f _= 2 × 10^-7 ^m^2^/s, *D*_B _= 4 × 10^-11 ^m^2^/s, *D*_T _= 6 × 10^-11 ^m^2^/s, and *μ *= 10^-3 ^Pas. It is also assumed that $\phi_{1}^{*}-\phi_{0}^{*}=0.001$, *β *= 3.4 × 10^-3^1/K, (*ρ*_*C*_)_f _= 4 × 10^6^J/m^3^, $T_{h}^{*}-T_{c}^{*}=1\;\text{K}$, and $T_{c}^{*}=300\;\text{K}$.

The parameter values given above result in the following representative values of dimensionless parameters: *Lb *= 1.5 × 10^3^, *Le *= 94, *Ln *= 5.0 × 10^3^, *Pr *= 5.0, *N*_*A *_= 5, *N*_*B *_= 7.5 × 10^-4^, *Pe *= 37, $\hat{\beta }=0.46$, ϖ = 17, *Ra *= 2.7 × 10^3^, *Rb *= 1.2 × 10^5^, *Rm *= 8.0 × 10^5^, and *Rn *= 2.3 × 10^3^. The values of *Ra *and *Rb *can be controlled by changing the temperature difference between the plates and the microorganism concentration, respectively, and *Rn *depends on nanoparticle concentrations at the boundaries.

**Figure 1:**
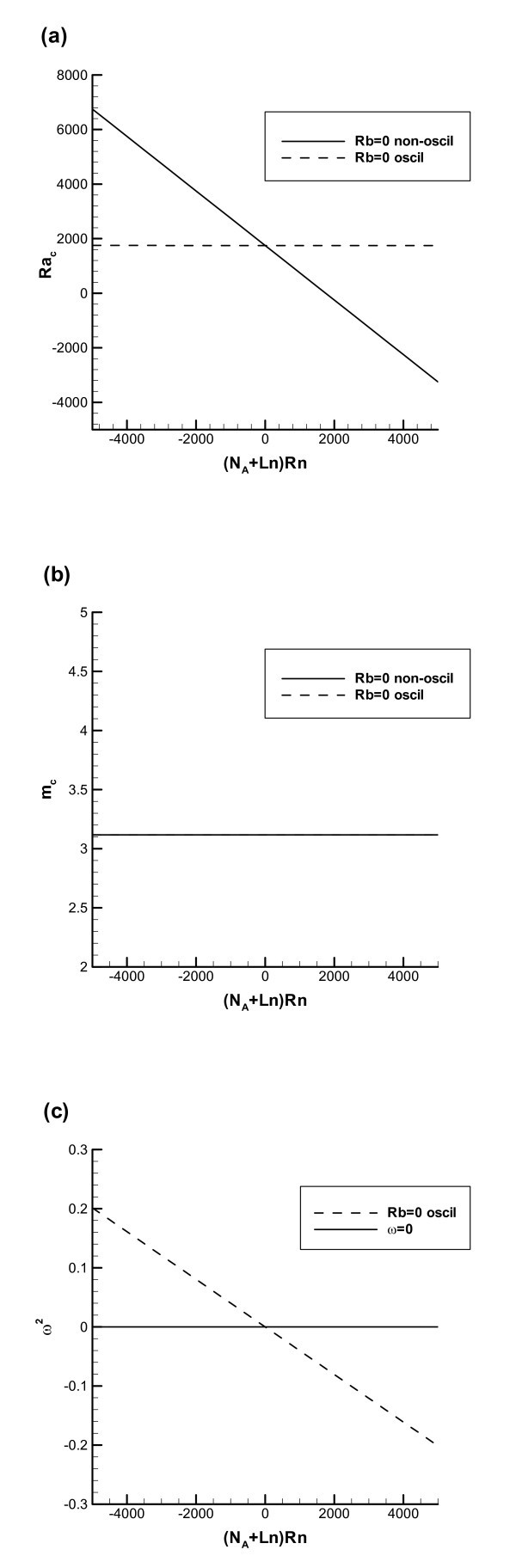
**The case of rigid upper and lower walls, *Rb *= 0 (no microorganisms)**: (**a**) Oscillatory and non-oscillatory instability boundaries in the (*Ra*_c_, *Rn*) plane. (**b**) Critical wavenumber in the (*Ra*_c_, *Rn*) plane. (**c**) Square of the oscillation frequency, *ω*^2^, versus the nanoparticle concentration Rayleigh number (for oscillatory instability to occur, *ω*^2 ^must be positive so that *ω *remains real).

For Figure 1a,b,c, the following values of dimensionless parameters are utilized: *Lb *= 1500, *Le *= 94, *Ln *= 5000, *Pr *= 5, *N*_A _= 5, *ϖ *= 17, and *Rb *= 0 (which corresponds to the situation with zero concentration of microorganisms). *Rn *is changing in the range between -1.2 and 1.2. In Figure 1a, the boundary for non-oscillatory instability (shown by a solid line) is obtained by setting *ω *to zero in Equation 54, solving this equation for *Ra *and then finding the minimum with respect to *m *of the right-hand side of the obtained equation. The boundary for oscillatory instability (shown by a dotted line) is obtained by the following procedure. Two coupled equations are produced by taking the real and imaginary parts of Equation 54. One of these equations is used to eliminate *ω*, and the resulting equation is then solved for *Ra*; the critical value of *Ra *is again obtained by calculating the minimum value that the expression for *Ra *takes with respect to *m*.

Figure 1a shows that for *Rb *= 0 the curve representing the instability boundary for non-oscillatory convection (solid line) is a straight line in the (*Ra*_c_, *Rn*) plane. *Rn *is defined in Equation 5 in such a way that positive *Rn *corresponds to a top-heavy nanoparticle distribution. Therefore, the increase of *Rn *produces the destabilizing effect and reduces the critical value of *Ra*. A comparison between instability boundaries for non-oscillatory (solid line) and oscillatory (dotted line) cases indicates that in order for the oscillatory instability to occur, *Rn *generally must be negative, which corresponds to a bottom-heavy (stabilizing) nanoparticle distribution. In this case the destabilizing effect of the temperature gradient (positive *Ra *corresponds to heating from the bottom) and destabilizing effect from upswimming of oxytactic microorganisms compete with the stabilizing effect of the nanoparticle distribution.

Figure 1b shows that the critical value of the wavenumber, *m*_c_, is independent of *Rn *and for the case displayed in Figure 1a (*Rb *= 0) is equal to 3.116; also, it is almost independent of the mode of instability (non-oscillatory versus oscillatory).

Figure 1c shows the square of the oscillation frequency, *ω*^2^, versus the nanoparticle concentration Rayleigh number, *Rn*. The value of *ω*^2 ^for the oscillatory instability boundary is obtained by eliminating *Ra *from the two coupled equations resulting from taking the real and imaginary parts of Equation 54 and solving the resulting equation for *ω*^2^. The solution is presented in terms of *ω*^2 ^rather than *ω *because the resulting equation is bi-quadratic in *ω*. For oscillatory instability to occur, *ω*^2 ^must be positive so that *ω *is real. Figure 1c shows that for *Rb *= 0 *ω *is real when *Rn *is negative.

**Figure 2:**
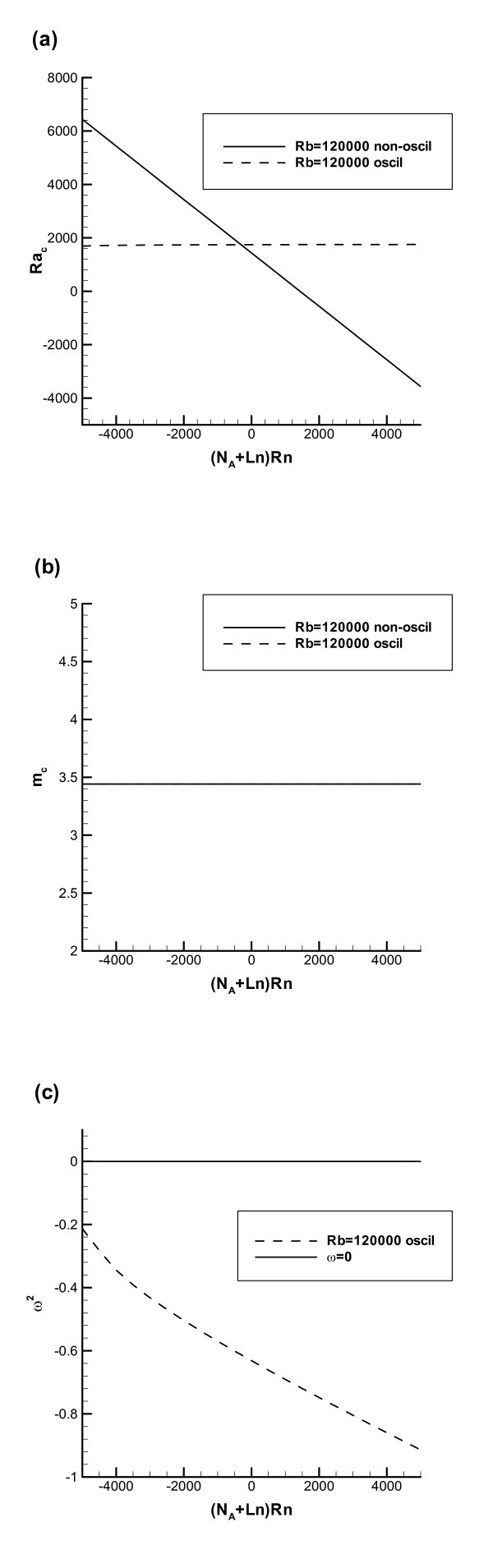
**Similar to Figure 1, but now with *Rb *= 120000**.

Figure 2a,b,c is computed for the same parameter values as Figure 1a,b,c, but now with *Rb *= 120000. Figure 2a,b,c thus shows the effect of microorganisms. By comparing Figure 2a with 1a, it is evident that the presence of microorganisms produces the destabilizing effect and reduces the critical value of *Ra*. For example, at (*N*_*A *_+ *Ln*) *Rn *= -5000 in Figure 1a the value of *Ra*_c _corresponding to the non-oscillatory instability boundary is 6750 and in Figure 2a the corresponding value of *Ra*_c _is 6437. At (*N*_*A *_+ *Ln*) *Rn *= 5000 in Figure 1a the value of *Ra*_c _corresponding to the non-oscillatory instability boundary is -3250 and in Figure 2a the corresponding value of *Ra*_c _is -3563. The destabilizing effect of oxytactic microorganisms is explained as follows. These microorganisms are heavier than water and on average they swim in the upward direction. Therefore, the presence of microorganisms produces a top-heavy density stratification and contributes to destabilizing the suspension.

The comparison of Figure 2b with 1b shows that the presence of microorganisms increases the critical wavenumber (in Figure 1b it was 3.116 and in Figure 2b it is 3.441).

Figure 2c brings an interesting insight. Apparently, if the concentration of microorganisms is above a certain value, the oscillatory mode of instability is not possible. Indeed, *ω*^2 ^in Figure 2c is negative for the whole range of *Rn *(-1.2 ≤ *Rn *≤ 1.2) used for computing this figure. This means that *ω *is imaginary and oscillatory instability does not occur for the value of *Rb *used in computing Figure 2.

### Rigid-free boundaries

For the case when the upper boundary is stress-free, the eigenvalue equation is

(56) $$F_{5}Ra+F_{6}Rn+F_{7}Rb-F_{8}=0$$

where functions *F*_5_, *F*_6_, *F*_7_, and *F*_8 _are given in the appendix [see Equations A10 to A13].

Again, to evaluate of the accuracy of the one-term Galerkin approximation in this case, the accuracy of Equation 56 is estimated for the case of non-oscillatory instability (which corresponds to *ω *= 0) for the situation when the suspension contains no microorganisms (*Rb *= 0) and no nanoparticles (*Rn *0). In this limiting case Equation 56 collapses to

(57) $$Ra=\frac{28\left(10+m^{2}\right)\left(4536+432m^{2}+19m^{4}\right)}{507m^{2}}$$

The right-hand side of Equation 57 takes the minimum value of 1139 at *m*_c_=2.670; the obtained value of *Ra*_c _is 3.48% greater than the exact value (1100.65) for this problem reported in [48]. The corresponding critical value of the wavenumber is 0.45% smaller than the exact value (2.682) reported in [48].

**Figure 3:**
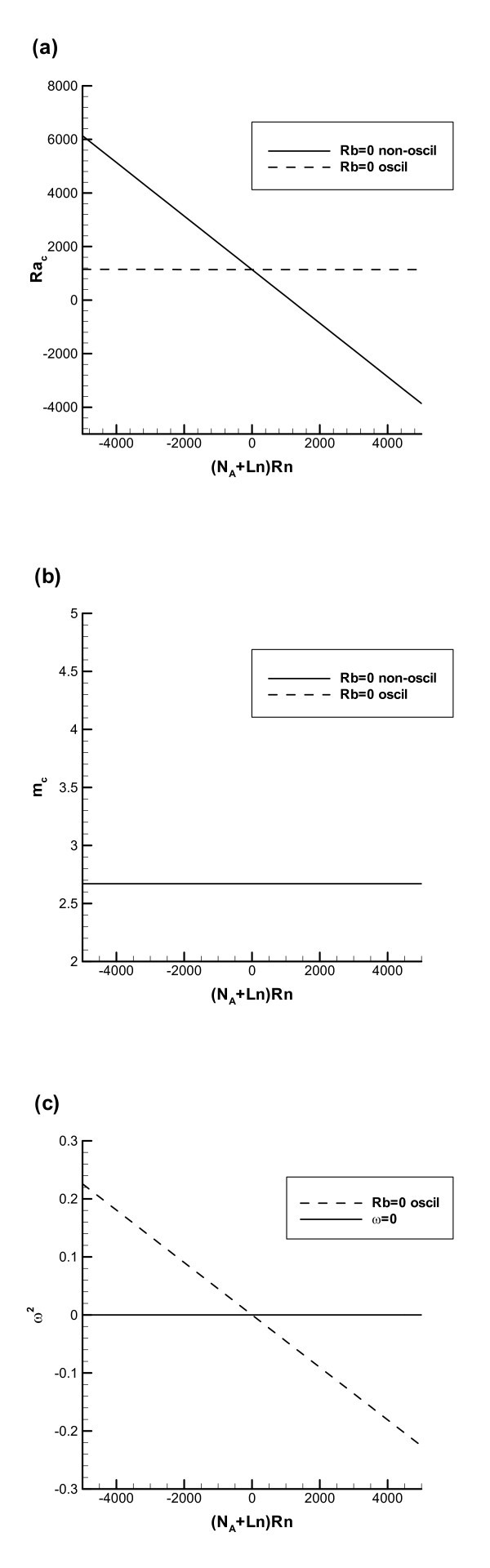
**The case of a rigid lower wall and a stress-free upper wall, *Rb *= 0 (no microorganisms)**: (**a**) Oscillatory and non-oscillatory instability boundaries in the (*Ra*_c_, *Rn*) plane. (**b**) Critical wavenumber in the (*Ra*_c_, *Rn*) plane. (**c**) Square of the oscillation frequency, *ω*^2^, versus the nanoparticle concentration Rayleigh number (for oscillatory instability to occur, *ω*^2 ^must be positive so that *ω *remains real).

**Figure 4:**
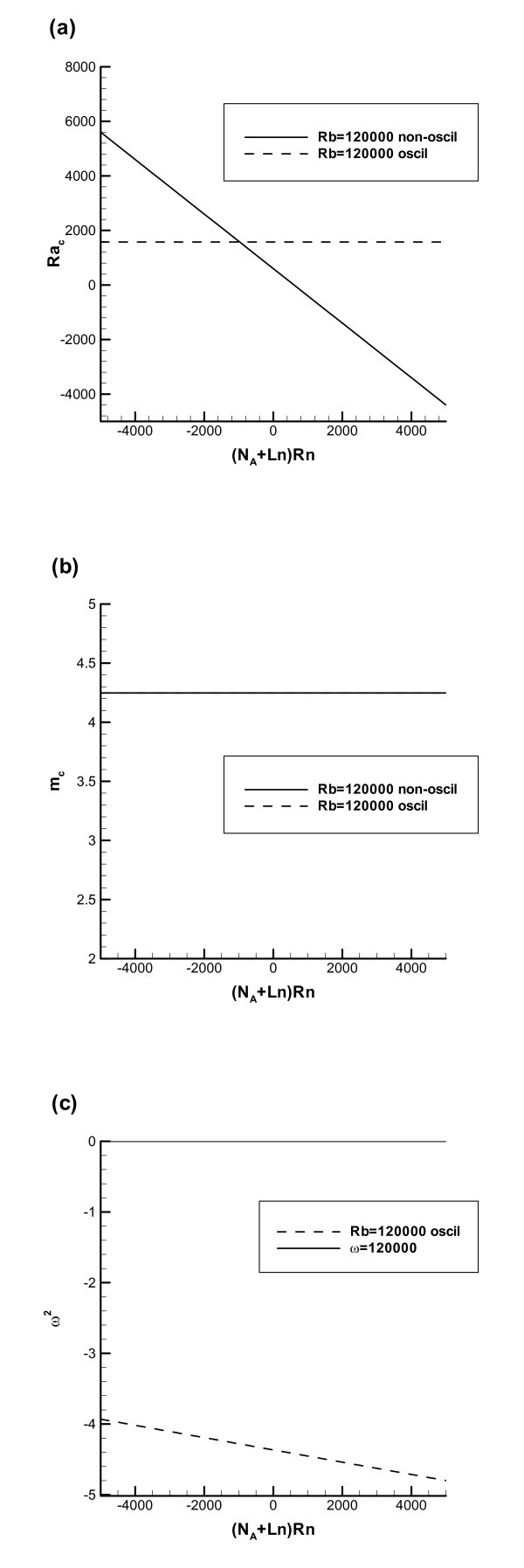
**Similar to Figure 3, but now with *Rb *= 120000**.

For Figures 3a,b,c and 4a,b,c, which show the results for the rigid-free boundaries, the same parameter values as for Figures 1 and 2 are utilized. Figure 3a, which is computed for *Rb *= 0 (no microorganisms), shows boundaries of non-oscillatory and oscillatory instabilities. This figure is similar to Figure 1a, but since now the case of the rigid-free boundaries is considered, the values of the critical Rayleigh number in Figure 3a are smaller than those in Figure 1a. Again, the comparison between the non-oscillatory and oscillatory instability boundaries indicates that in order for oscillatory instability to occur *Rn *must be negative; in this case at the instability boundary the effect of the nanoparticle distribution is stabilizing and the effect of the temperature gradient is destabilizing; the presence of these two competing agencies makes the oscillatory instability possible.

The critical wavenumber shown in Figure 3b (*m*_c _= 2.670) is smaller than the corresponding critical wavenumber for the rigid-rigid boundaries shown in Figure 1b. Again, it is independent of *Rn *and almost independent of the mode of instability (non-oscillatory versus oscillatory).

Figure 3c, similar to Figure 1c, shows that *ω *is real when *Rn *is negative, which means that for negative values of *Rn *oscillatory instability is indeed possible.

Figure 4a,b,c shows the results for rigid-free boundaries computed with *Rb *= 120000, meaning that the difference with Figure 3a,b,c is the presence of microorganisms. As in the case with rigid-rigid boundaries, the presence of microorganisms produces a destabilizing effect and reduces the critical value of the Rayleigh number (compare Figures 4a and 3a).

Also, the presence of microorganisms increases the critical value of the wavenumber (compare Figures 4b and 3b).

Figure 4c again shows that for the range of Rn used for this figure the presence of microorganisms makes the oscillatory mode of instability impossible (corresponding values of *ω *are imaginary).

## Conclusions

The possibility of oscillatory mode of instability in a nanofluid suspension that contains oxytactic microorganisms is investigated. Since these microorganisms swim up the oxygen concentration gradient, toward the free surface (which is open to the air), and they are heavier than water, they always produce the destabilising effect on the suspension. The destabilizing effect of microorganisms is larger if their concentration in the suspension is larger. The concentration of microorganisms is measured by the bioconvection Rayleigh number, *Rb*, which by definition is always non-negative (the zero value of *Rb *corresponds to a suspension with no microorganisms). The increase of *Rb *thus destabilizes the suspension. It is also shown that the presence of microorganisms increases the critical wavenumber.

The effect of the temperature distribution can be either stabilizing (heating from the top, negative thermal Rayleigh number *Ra*) or destabilizing (heating from the bottom, positive *Ra*). The effect of nanoparticles can also be stabilizing (bottom-heavy nanoparticle distribution, negative nanoparticle concentration Rayleigh number *Rn*) or destabilizing (top-heavy nanoparticle distribution, positive *Rn*).

The results obtained in this article indicate that in order for the oscillatory instability to occur, *Rn *generally must be negative, which corresponds to a bottom-heavy (stabilizing) nanoparticle distribution. In this case the destabilizing effect of the temperature gradient (positive *Ra*) and destabilizing effect from upswimming of oxytactic microorganisms compete with the stabilizing effect of the nanoparticle distribution.

In order for the oscillatory mode of instability to occur, the dimensionless oscillation frequency, *ω*, must be real. Since increasing *Rb *pushes *ω*^2 ^to negative values, oscillatory instability is possible only if the concentration of microorganisms is below a certain value.

The results for the rigid-rigid and rigid-free boundaries are similar, but the critical Rayleigh number for the rigid-free boundaries is smaller. The critical wavenumber for the rigid-free boundaries can be either smaller or larger, depending on the concentration of microorganisms. For *Rb *= 0 the critical wavenumber is smaller for the rigid-free boundaries but for *Rb *= 120000 it is larger than for the rigid-rigid boundaries.

## Appendix

The functions *F*_1_, *F*_2_, *F*_3_, and *F*_4 _defining the eigenvalue equation for the layer with the rigid-rigid boundaries [given by Equation 54] are

(A1) F1=1265Lb m2Prϖ(10+m2+i Lnω)[−15Le(I3+A12I4m2)(5+2α) +15I2(5+2m2+2i Leω)+4Le(15+2α(5+α))ϖ(m2+i Lbω)(5+2m2+2i Leω)]

(A2) F2=1265Lbm2Prϖ[(10+m2)(Ln+NA)+i Lnω]{−15Le(I3+A12I4m2)(5+2α) +15I2(5+2m2+2i Leω)+4Le(15+2α(5+α))ϖ(m2+i Lbω)(5+2m2+2i Leω)]

(A3) F3=294A1m2Pr(14+5α)(10+m2+i ω)(10+m2+i Lnω) ×[15I5Le(I3+A12I4m2)+32A12I1Lbϖ(5+2m2+2i Leω)]

(A4) F4=39215Lbϖ(10+m2+i ω)(10+m2+i Lnω)[(504+24m2+m4)Pr+i(12+m2)ω] ×{−15Le(I3+A12I4m2)(5+2α)+15I2(5+2m2+2i Leω)+4Le[15+2α(5+α)] ×ϖ(m2+i Lbω)(5+2m2+2i Leω)}

The integrals *I*_1 _to *I*_5 _in Equations A1 to A4 are functions of *Le *and *ϖ*. The expressions for these integrals for the rigid upper boundary case are given below:

(A5) $$I_{1}=\int_{0}^{1}{\left(-1+z\right)}^{2}z^{2}\left(1-\frac{1}{2}\left(-2+z\right)z\alpha \right)\csc^{3}\left[A_{1}\left(1-z\right)\right]\sin^{4}\left[\frac{1}{2}A_{1}\left(1-z\right)\right]\;\text{d}z$$

(A6) I2=∫01{(1−12(−2+z)zα)[4Leαϖ+A12Le(2−(−2+z)zα)ϖsec2[12A1(−1+z)] −4A1Le(−1+z)αϖtan[12A1(−1+z)]]}

(A7) I3=∫01{(1−12(−2+z)zα) ×{−2A12ϖsec2[12A1(−1+z)]−2A13(−1+z)ϖsec2[12A1(−1+z)]tan[12A1(−1+z)]}dz

(A8) $$I_{4}=\int_{0}^{1}\left(-2+z\right)z\left(1-\frac{1}{2}\left(-2+z\right)z\alpha \right)\varpi \sec^{2}\left[\frac{1}{2}A_{1}\left(-1+z\right)\right]\text{d}z$$

(A9) $$I_{5}=\int_{0}^{1}\left(-2+z\right){\left(-1+z\right)}^{2}z^{3}\tan\left[\frac{1}{2}A_{1}\left(1-z\right)\right]\text{d}z$$

The functions *F*_5_, *F*_6_, *F*_7_, and *F*_8 _defining the eigenvalue equation for the layer with the rigid-free boundaries [given by Equation 56] are

(A10) F5=23665Lbm2Prϖ(10+m2+i Lnω){30I^2(5+2m2+2i Leω)+Le[−30I^3(5+2α) −15A12I^4m2(5+2α)+4(15+2α(5+α))ϖ(m2+i Lbω)(5+2m2+2i Leω)]}

(A11) F6=23665Lbm2Prϖ((10+m2)(Ln+NA)+i Lnω){30I^2(5+2m2+2i Leω)+Le[−30I^3(5+2α) −15A12I^4m2(5+2α)+4(15+2α(5+α))ϖ(m2+i Lbω)(5+2m2+2i Leω)]}

(A12) F7=147A1m2Pr(126+41α)(10+m2+i ω)(10+m2+i Lnω) ×[30I^3I^5Le+A12{15I^4I^5Lem2+4I^1Lbϖ(5+2m2+2i Leω)}]

(A13) F8=39215Lbϖ(10+m2+i ω)(10+m2+i Lnω)[(4536+432m2+19m4)Pr+i(216+19m2)ω] ×{30I^2(5+2m2+2i Leω)+Le[−30I^3(5+2α)−15A12I^4m2(5+2α)+4(15+2α(5+α)) ×ϖ(m2+i Lbω)(5+2m2+2i Leω)]}

The integrals ${\hat{I}}_{1}$ to ${\hat{I}}_{5}$ in Equations A10 to A13 are functions of *Le *and *ϖ*. The expressions for these integrals for the stress-free upper boundary case are given below:

(A14) $${\hat{I}}_{1}=\int_{0}^{1}\left(-1+z\right)z\left(1+z-2z^{2}\right)\left(1-\frac{1}{2}\left(-2+z\right)z\alpha \right)\sec^{2}\left[\frac{1}{2}A_{1}\left(-1+z\right)\right]\tan\left[\frac{1}{2}A_{1}\left(-1+z\right)\right]\text{d}z$$

(A15) I^2=∫01[1−12(−2+z)zα]{2Leαϖ+12A12Le[2−(−2+z)zα]ϖsec2[12A1(−1+z)] −A1Le(−1+z)αϖsec2[12A1(−1+z)]tan[12A1(−1+z)] −A1Le(−1+z)αϖcos[A1(−1+z)]sec2[12A1(−1+z)]tan[12A1(−1+z)]}dz

(A16) I^3=∫01[1−12(−2+z)zα]{−A12ϖsec2[12A1(−1+z)]−A13(−1+z)ϖsec2[12A1(−1+z)] ×tan[12A1(−1+z)]dz

(A17) $${\hat{I}}_{4}=\int_{0}^{1}\left(-2+z\right)z\left[1-\frac{1}{2}\left(-2+z\right)z\alpha \right]\varpi \sec^{2}\left[\frac{1}{2}A_{1}\left(-1+z\right)\right]\text{d}z$$

(A18) $${\hat{I}}_{5}=\int_{0}^{1}\left(-2+z\right){\left(-1+z\right)}^{2}z^{2}\left(1+2z\right)\tan\left[\frac{1}{2}A_{1}\left(1-z\right)\right]\text{d}z$$

## Competing interests

The author declares that he has no competing interests.

## Authors' contributions

AVK carried out all the work regarding the development of the model, performing simulations, writing and revising the paper and approving the final manuscript.
